# Folate Enrichment of Whole-Meal Spaghetti Using Durum Wheat Debranning Fractions

**DOI:** 10.3390/foods12132575

**Published:** 2023-06-30

**Authors:** Valentina Di Nardo, Elisa De Arcangelis, Maria Cristina Messia, Stefania Ruggeri, Emanuele Marconi

**Affiliations:** 1 Dipartimento Agricoltura, Ambiente e Alimenti (DiAAA), Università degli Studi del Molise—Via F. De Sanctis, 86100 Campobasso, Italy; 2 Department of Science and Technology for Sustainable Development and One Health, Università Campus Bio-Medico di Roma, Via Alvaro del Portillo 21, 00128 Rome, Italy; 3 Council for Agricultural Research and Economics (CREA), Research Centre for Food and Nutrition, Via Ardeatina 546, 00178 Rome, Italy

**Keywords:** debranning, whole-meal, folate, raw and cooked pasta, folate retainment

## Abstract

Durum wheat debranning fractions (fine and coarse bran) were obtained and included as an ingredient in the formulation of whole-meal spaghetti to study their chemical–nutritional characteristics, in particular folate levels and sensorial properties. Experimental raw pasta had a higher folate content (40.5 µg/100 g) than commercial whole-meal pasta (28.3 µg/100 g), meeting the requirements for the health claim on folate (Reg. EU 432/2012) and for the nutritional claim on dietary fiber. After cooking, folate retention in pasta formulated with coarse bran was 80% and scored an overall “good” sensorial acceptability. Results indicate that whole-meal pasta formulated with folate-rich debranning fractions may represent a natural functional food that, integrated into the diet, could improve the health status of the population.

## 1. Introduction

In recent years, the demand for whole grain meals/foods has increased due to consumers’ awareness of their health benefits [1]. Several epidemiological studies suggest a clear inverse relationship between the consumption of whole grain products and the risk of non-communicable diseases such as cardiovascular diseases, type 2 diabetes (T2D), and colorectal cancer [2]. These beneficial effects are probably due to the combined action of dietary fiber, micronutrients (minerals and vitamins), and other bioactive compounds (i.e., polyphenols, tocols, alkylresorcinols, phytosterols), which are largely preserved in whole grain products compared to refined ones [3]. Many studies have demonstrated the positive effects on human health of wheat bran and its bioactive components in the prevention and treatment of some chronic diseases such as diabetes, cardiovascular disease, obesity, gastrointestinal disease, and colon and breast cancers [4,5]. Beyond the evidence from in vitro models, animal models, and cell lines, it would also be important to broaden knowledge on the beneficial outcome of wholegrain intake on healthy people or those suffering from different diseases. For instance, arabinoxylan-rich fiber can improve blood glucose control in patients with type II diabetes [6]. A prospective cohort study conducted on 161,737 US women and a systematic review [7] demonstrated a stronger inverse association between bran intake and risk of type 2 diabetes.

Among cereal-based products, pasta represents an important carrier of these beneficial compounds for the population [8,9,10,11,12], given its popularity, relatively low cost, and long shelf life. Unfortunately, the consumption of whole grain products, which are much more nutritious, is still low even in countries including Italy [1]. It would be desirable to be able to formulate a new kind of whole grain pasta naturally enriched in some vitamins such as folate, which many population groups are deficient in, and which are necessary for general health [13,14,15]. Folate deficiency is associated with several diseases, including megaloblastic anemia, neural tube defects (NTDs) [16], intrauterine growth restriction and preterm birth [17], cardiovascular diseases [18], and neurodegenerative diseases (i.e., Alzheimer’s, dementia) [19]. Folates are mainly localized in the aleurone layer of grain [20,21] and whole-meal products can be an important source of folate in the diet [22].

Fractionation/recombination technologies applied to cereals are widespread techniques to obtain flours enriched with vitamins or bioactive molecules that can enhance the nutritional potential of a new product [23,24,25]. Wheat debranning, for example, consists of friction/abrasion operations applied to the kernel prior to milling to remove bran layers. Advantages include the improvement of the refining rate, yield, and safety of semolina and the simplification of the milling diagram [26,27,28]. In the literature, previous studies incorporated durum wheat debranning fractions in cereal-based formulations, enhancing the content of healthy compounds, along with conducting an assessment of the technological profile of the final product [8,28]. In particular, folate-rich fractions can be obtained with the use of the debranning process of durum wheat [25,29]; these raw materials represent an important starting point for the development of whole-meal pasta, the nutritional properties of which are improved.

The aim of the study was to evaluate durum wheat debranning fractions as an ingredient for folate enrichment of whole-meal pasta, assessing the nutritional and organoleptic acceptability of the final product.

## 2. Materials and Methods

### 2.1. Debranning/Milling

**Figure 1:**
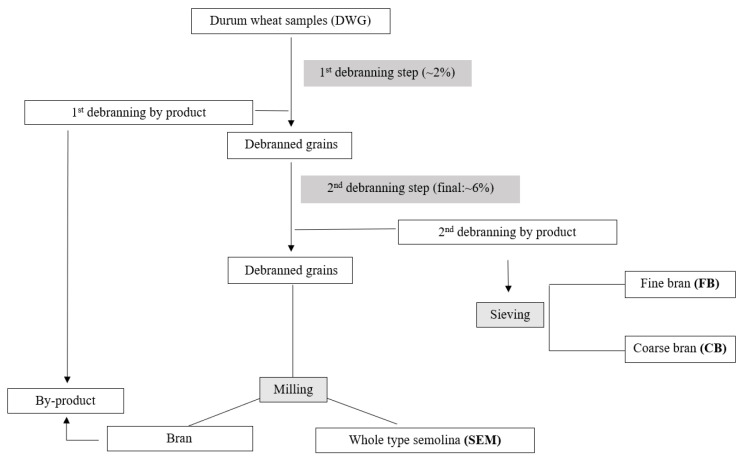
Schematic representation of the milling process and the obtained fractions.

Durum wheat grain blends (DWG) (*Triticum turgidum* L. subsp. *durum*) were conditioned to about 17% moisture and debranned in two sequential steps using a pilot plant. Firstly, the grain was debranned at about 2% and the by-product was discarded; then, the process was repeated to remove about 4% of the outer layers of the debranned kernel (corresponding to a total removal of ~6% of the initial kernel weight). The debranning by-product obtained after the second debranning step was sorted in a plansichter equipped with six sieve stacks of different mesh sizes to obtain a fine bran fraction (FB) and a coarse bran fraction (CB). Debranned grain was subsequently ground in an MLU 202 mill (Bühler, Uzwill, Switzerland) consisting of three break and three reduction rolls, six steel screens, and a small-scale purifier. Whole-type semolina was used for pasta formulation. A schematic graphic representation of the milling process is illustrated in Figure 1. All samples were stored at +4 °C before analysis.

### 2.2. Particle Size Distribution

Particle size distribution of debranning fractions was determined according to the Italian standard UNI 10873:2000 [30] by a sieve shaker equipped with a stack of 7 sieves with different size meshes—630, 560, 400, 355, 250, 180, 100 μm—(Retsch GmbH, Italy). The sample was weighed (100 g) and sieved for 5 min.

### 2.3. Pasta Making

Fine bran and coarse bran were mixed with semolina following the different formulations, as reported in Table 1. After folate determination in raw materials, the formulations were considered to achieve the nutrition and health claims related to folate, according to EC Regulation No 1924/2006 and EU Regulation No 432/2012 [31,32], and to fall within the legal limits set for ash for durum wheat whole-meal semolina pasta by the Italian Presidential Decree No 187/2001 [33]. Whole-meal pasta samples (spaghetti shape) were produced in two different pasta-making trials through an experimental pasta-making pilot plant, composed of a press and a dryer (NAMAD, Rome, Italy). The press (capacity 10–20 kg) was equipped with a vacuum mixing and extruding system, a die, and a water-cooling jacket for the barrel and the extrusion head (to maintain a constant extrusion temperature lower than 50 °C). Semolina and fine and coarse bran fractions were mixed with cold tap water in a pre-mixing chamber for 20–30 min, then the doughs were transferred to the vacuum mixing chamber and extruding system equipped with a bronze die. Extruded spaghetti was dried by applying drying cycles at low temperatures (<60 °C).

### 2.4. Commercial Pasta Sampling

Eight whole-meal pasta samples (CWP_1–8_) (spaghetti shape) and two semolina pasta samples (CSP_1–2_) (spaghetti shape) of different brands were purchased in local supermarkets. All the pasta samples were ground using a refrigerated laboratory mill (model IKA A10-IKA Werke GmbH & Co. KG, Staufen, Germany) and stored at +4 °C until analysis.

### 2.5. Spaghetti Cooking

Spaghetti samples were cooked in tap water (pasta: water ratio of 1:10) until the optimal cooking time (OCT), according to the International Standard ISO 7304-1:2016 [34]. Cooked samples were drained and cooled for about 10 min at room temperature and freeze-dried in a VirTis Genesis 25SES Pilot Lyophilizer (VirTis Co., Inc., Gardiner, NY, USA) for chemical analyses. The freeze-dried cooked samples were ground with a refrigerated laboratory mill (model IKA A10-IKA Werke GmbH & Co. KG, Staufen, Germany), analyzed for their residual moisture content, and stored at −20 °C before analysis.

### 2.6. Color

The color determination was performed using a colorimeter model CR300 (Minolta Italia, S.p.A., Milan, Italy). The results are expressed in terms of CIE (Commission Internationale de l’Eclairage) 1976 L* a* b* color space parameters, where L* describes lightness, a* is redness, and b* is yellowness.

### 2.7. Chemical Analysis

Moisture and ash contents were analyzed according to ICC Standard Methods No. 110/1 [35] and International Standard ISO 2171:2007 [36], respectively. Protein content was determined with the Kjeldahl method according to ICC Standard Method No. 105/2 [35], using a conversion factor of 6.25. The lipid content was determined by acidic hydrolysis following the AOAC Official Method 922.06 [37]. The total dietary fiber was determined using the enzymatic assay kit (Megazyme International Ltd., Bray, Ireland), in accordance with the AOAC Official Method 985.29 [37].

### 2.8. Total Folate Content and Apparent Folate Retention Determination

Total folate content was determined using the microbiological assay kit (VitaFast^®^ Folic Acid microbiological microtiter plate test to quantitate folic acid) distributed by R-Biopharm AG, Darmstadt, Germany. This procedure is recognized by international standardization institutes, e.g., EN 14131:2003 and AOAC 960.46 (Method 2004.05). Briefly, 1 g sample was extracted in 40 mL phosphate buffer (0.05 mol/L, 0.1% ascorbate, pH 7.2) and 10 mg chicken pancreatin (VitaFast^®^ Chicken Pancreatin (γ-Glutamylhydrolase)) which was purchased from R-Biopharm AG, Darmstadt, Germany. The extracts were diluted depending on the concentration range and then inoculated into the wells of a 96-well microtiter plate coated with *Lactobacillus rhamnosus*, together with the culture medium. After an incubation period of 48 h in the dark at 37 °C, the turbidity was measured with a microtiter plate reader at 620 nm. The total folate content of the samples was calculated by comparison with a calibration curve constructed with the folic acid standard. The certified reference material BCR-121 (whole-meal flour; action limit: 50 ± 7 μg/100 g d.m.) obtained from the Institute for Reference Materials and Measurements (Geel, Belgium) was analyzed in each set of samples as a quality control sample. The average experimental folate content obtained for the certified reference material was 49 ± 4 μg/100 g of d.m.

Apparent folate retention (AR) was calculated on cooked pasta, defined by Murphy et al. (1975) [38] as follows:

$$\%\;\mathrm{AR}=\frac{folate\;content\;per\;g\;of\;cooked\;spaghetti\left(dry\;basis\right)}{folate\;content\;per\;g\;of\;raw\;spaghetti\;(dry\;basis)}\times 100$$

### 2.9. Furosine Determination

The determination of furosine was carried out according to the method proposed by Resmini et al. (1990) [39]. A sample amount corresponding to 30–70 mg of proteins was hydrolyzed under nitrogen with 8 mL of 8 N HCl at 110 °C for 23 h. The hydrolysate was filtered on a filter paper Whatmann no. 4. The filtrate (0.5 mL) was purified on a Sep-Pak C18 cartridge (Waters Corporation, Milford, MA, USA) and analyzed by HPLC (Dionex, Sunnyvale, CA, USA) equipped with a furosine-dedicated column (Grace, Reading, Berkshire, UK) and a photodiode array detector (Dionex, Sunnyvale) set at 280 nm wavelength. The quantification of furosine was achieved using an external standard purchased from Neosystem Laboratoire (Strasbourg, France).

### 2.10. Cooking Quality and Sensory Evaluation

Experimental whole-meal pasta samples cooked at the optimal cooking time (OCT) were subjected to sensory analysis according to the International Standard ISO 7304-2:2008 [40]. A trained panel of 10 individuals conducted the sensory analysis on the cooked whole-meal pasta samples in a sensory room. The samples were provided in white dishes immediately after cooking and in a randomized order. The panelists were asked to assess the following sensory attributes: liveliness by manual handling, firmness by chewing, and starch release by manual handling. The final judgment was expressed on a scale from 10 to 100 and the total score was weighed for the individual sensory attributes considered.

### 2.11. Statistical Analysis

All experiments were performed at least in triplicate and the data were reported as means ± standard deviation (SD). One-way ANOVA with Scheffé’s post-hoc test was conducted to determine significant differences between means (*p* < 0.05) using SPSS software (version 22.0, IBM SPSS Statistics, Armonk, NY, USA).

## 3. Results

### 3.1. Particle Size Distribution and Folate Content of Durum Wheat Debranning Fractions

**Figure 2:**
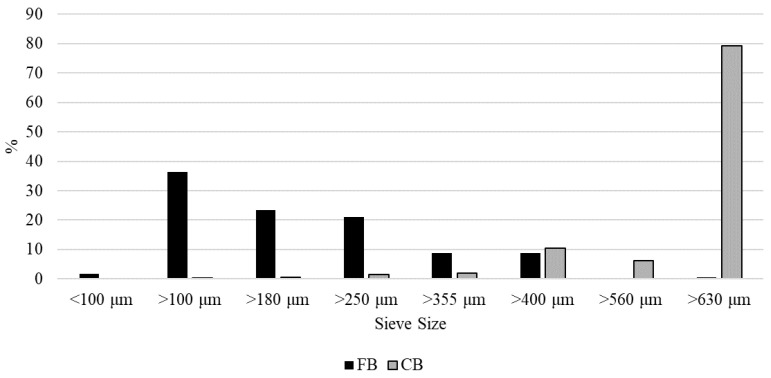
Average particle size distribution of bran fractions. FB, fine bran; CB, coarse bran.

The particle size distribution of the debranning fractions is shown in Figure 2. The fine bran particles are mainly distributed between 100 and 250 μm (approx. 60%), unlike the coarse bran particles in which the particle distribution was greater than 630 μm (79%).

Table 2 shows the ash, protein, and folate distribution in the grain, semolina, and debranning products. Folate accumulation in grain reached 60.3 µg/100 g d.m. The fine bran (FB) fractions had a total folate content of 218.9 µg/100 g d.m, while for coarse bran, (CB) it reached 204.9 µg/100 g d.m. As expected, the semolina was characterized by a drop of folate content compared to grain, reaching 25.8 µg/100 g f.w. (30.2 µg/100 g d.m.), while in Ruggeri et al. (2022) [25], commercial semolina displayed values of 35.0–40.2 µg/100 g f.w. The results confirm the greater localization of folate in the outer cell layers, especially in the aleurone layer, and in the germ of the wheat grain, coupled with a substantial folate depletion in the endosperm tissues [21,41]. These results are consistent with previous studies in grains, despite the large variability in folate accumulation, which may be due to environmental and genetic factors [42]. In durum wheat, Piironen et al., (2008) [20] found the range of folate to be 63.7–89.1 µg/100 g d.m., unlike Giordano et al., (2015) [29], who reported a value of approximately 112 µg/100 g d.m. In the same study, fractions obtained from wheat pearling 0–5% concentrated 205.0 µg/100 g d.m. folate in common wheat, and 267.0 µg/100 g d.m. in durum wheat, while with 5–10% pearling, values reached 189.5 µg/100 g d.m. and 265.1 µg/100 g d.m., respectively. In barley, Ruggeri et al. [25] reported a higher content of folate in pearling by-products compared to kernel, reaching the highest level (221.7 µg/100 g) in the second pearling step (yield: 19.3%).

Overall, according to our results, both the coarse and fine products of the second step of durum wheat debranning were naturally enriched in folate and can serve as optimal raw material to increase the folate levels in derived foods.

### 3.2. Chemical–Nutritional Composition and Colorimetric Indices of Experimental Whole-Meal Pasta

The composition of the experimental whole-meal spaghetti is shown in Table 3. Protein content was 15.0% and 15.2% in WP-FB and WP-CB, respectively, while the inclusion of coarse bran in the formulation resulted in a higher accumulation of dietary fiber, 8.3 g/100 g f.w., compared to pasta formulated with fine bran (6.5 g/100 g f.w.). Overall, the pasta samples had a fiber content greater than 6 g/100 g and, therefore, could be labeled with the claim “High fibre” according to the European Regulation No 1924/2006 [31] on nutrition and health claims on food. Studies have shown the significant contribution of bioactive compounds occurring in bran to multiple physiological effects, especially for healthy gastrointestinal function. A higher intake of dietary fiber is beneficial for microbiota diversity and/or abundance [43,44], specifically in relation to the arabinoxylan fraction [45], also providing an enhancement of the intestinal barrier [44]. Furthermore, interesting evidence is emerging about the possible role of folate in the regulation of immunoregulatory control of the gastrointestinal tract microbiome [46].

As shown in Table 3, the furosine level was equal to 224 mg/100 g protein in the WP-FB sample, and 245 mg/100 g in the WP-CB sample. Marti et al., (2017) [47] found furosine values for whole-meal pasta samples that varied over a wide range between 229 and 836 mg/100 g protein. This variability can be attributed to the drying diagram, as well as to the characteristics of the raw materials, such as the presence of damaged starch, the amylase activity, and the content of reducing sugars and proteins [47,48,49]. Additionally, no significant differences were found for the colorimetric indexes L* and b* (Table 3). It is well known that high-temperature drying (>65 °C) improves the cooking properties of pasta, especially when semolina has low/weak gluten [50]. Nevertheless, whole-meal pasta made with high-temperature drying may be perceived as more bitter and with weaker appearance, mechanical properties, and firmness than products obtained with low-temperature drying [51].

The results of this study, combined with previous evidence, show that the use of durum wheat debranning fractions and the adoption of a low drying temperature enables the production of whole-meal pasta with low heat/nutritional damage, while also preserving positive impacts on consumer perception.

### 3.3. Total Folate Content in Raw Experimental and Commercial Pasta Samples

**Figure 3:**
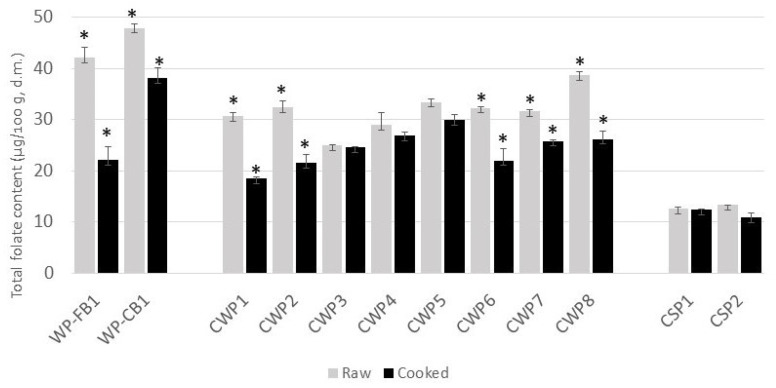
Folate variation in raw and cooked pasta samples (mean values ± SD). The asterisk represents statistically significant differences (*p* < 0.05) in folate content (% d.m.) between raw and cooked samples. WP-FB, WP-CB, experimental whole-meal pasta; CWP1-CWP8, commercial whole-meal pasta; CSP1 CSP2, commercial semolina pasta.

As reported in Table 3, the experimental samples of whole-meal raw pasta had a total folate content of 37.8 and 43.1 μg/100 g f.w. in WP-FB and WP-CB, respectively. Further, Figure 3 also shows the folate content of the experimental and commercial raw and cooked spaghetti samples (% d.m.). It is noteworthy that the experimental pasta obtained in this work had a higher folate level (% d.m.) than the average commercial whole-meal pasta (+42%). In fact, the mean folate value in commercial whole-meal pasta samples was 31.6 μg/100g d.m., ranging between 25.0 μg/100 g d.m. and 38.7 μg/100 g d.m. The large variability in commercial whole-meal pasta could be ascribed to the differences in the folate content of raw materials and milling techniques adopted to obtain whole-meal flours [52] which, consequently, determine the placing on the market of whole-meal products with different chemical and nutritional characteristics. As previously discussed, starchy endosperm tissues of grains are substantially depleted of folate compared to bran layers; thus, the folate content in whole-meal pasta samples (experimental and commercial) (Figure 3) exceeded the value found in commercial semolina pasta, which had an average total folate content of 13.0 μg/100 g d.m. The results of this study evidenced that the total folate values found in our whole-meal pasta samples (37.8 μg/100 g f.w. and 43.1 μg/100 g f.w. in WP-FB and WP-CB, respectively) were lower than those reported by Hirawan and Beta (2014) [53], of 57 μg/100 g, and Ruggeri et al., (2022) (50.5–61.1 μg/100 g f.w.) [25]. However, as previously mentioned, folate levels in cereal-based products are influenced by agronomic and environmental factors related to raw materials, as well as by milling and processing techniques.

According to European regulations [31,32,54], nutrition and health claims on folate can be provided if the food product covers at least 15% of the nutrient reference value (200 μg). Experimental whole-meal pasta samples met these requirements and achieved a folate content greater than 30 μg/100 g, and thus were allowed to boast the relevant nutrition (“source of folate”) and health claims. On the other hand, among the commercial samples, only CWP_5_ and CWP_8_ met the requirements for health claims on folate.

### 3.4. Folate Content and Retention in Experimental and Commercial Pasta Samples after Cooking

To evaluate the impact of cooking on folate retention, this index (apparent folate retention—AR) was calculated in the pasta samples (experimental and commercial), considering the folate content, before and after cooking, expressed on a dry matter basis (Figure 3). In all pasta samples, the total folate content was lower in the cooked pasta than in the raw pasta, with folate losses in WP-FB and WP-CB being 47.5% and 20.4%, respectively. Given the water solubility of folate, losses of these nutrients during boiling may be due to both thermal degradation and leaching into the cooking water [55,56]. Nonetheless, the experimental whole-meal pasta had higher folate levels than the commercial pasta after cooking.

The experimental whole-meal pasta retained folate in the measure of 52% in WP-FB and 80% in WP-CB, while the average value in commercial samples was 78%. However, the highest folate retention was found in semolina pasta samples, with results equal to 89%. In the scientific literature, few studies have evaluated the effect of the cooking process on folate retention in pasta. Ranhotra et al., (1985) [57] calculated the “true” retention of folate in semolina pasta samples, also taking into account the loss of solids during cooking, and found that the cooked samples retained a significant amount of folate, on average equal to 79% for spaghetti and 77% for macaroni. Liang et al., (2020) [58] found high folate retention in noodles samples after cooking, equal to an average of 78%, and an average loss of folate of 13% from boiling degradation. Similarly, Bui and Small (2007) [59] found that boiling the noodles resulted in an average loss of folate ranging from 13% to 30% for white and yellow noodles, while for instant noodles, the loss of these nutrients was very low, and between 4% and 6%. Pasta produced with barley pearling by-products had an average folate retention of 68.5% according to Ruggeri et al., (2022) [25]. In the same study, whole-meal commercial pasta samples displayed lower folate retention (49%) compared to our data. Remarkably, WP-CB was able to retain an amount of folate greater than 30 μg even after cooking, thus potentially maintaining the health claim for the raw pasta after domestic preparation. In contrast, in commercial pasta samples CWP5 and CWP8, the folate losses do not allow the health claim to be maintained after cooking.

These results suggest that the inclusion in the diet of whole-meal pasta produced using the folate-rich durum wheat debranning fractions could substantially contribute to meeting dietary reference values for folate intake (250 µg/day [9]).

### 3.5. Sensorial Properties of Cooked Spaghetti Samples

The use of coarse bran in the formulation resulted in a shorter cooking time (Table 3), which is probably due to a greater physical disruption of the gluten matrix by coarser bran particles, leading to increased water absorption. Our evaluation showed that the whole-meal pasta made with fine bran had slightly better sensory properties (overall score 87) than the whole-meal pasta made with coarse bran (overall score 82). Firmness was positively judged for both samples, while also presenting strands of pasta separated from each other (liveliness) and with no starchy residues (starch release). Additionally, the panelists perceived the WP-CB samples as having an intense and fragrant flavor of whole-meal semolina and a slightly woody taste. These results are consistent with those of Steglich et al., (2015) [60], as bran particle size did not significantly influence the sensory attributes of liveliness and firmness, while whole-meal spaghetti with larger bran particles had a more intense flavour than whole-meal spaghetti with smaller bran particles.

## 4. Conclusions

The incorporation of durum wheat debranning products—fine bran, coarse bran—in whole-meal pasta contributed to a marked improvement in the folate content of the final product, by an average of 40.5 μg/100 g. The pasta products can be labeled as “Source of folate” and boast the related health claims. The study of folate retention after cooking showed that samples formulated with coarse bran retained the highest amount of folate (80%), which opened up the possibility of also claiming the health effect on folate after preparation. The nutritional advantages for healthy adults also included a high availability of dietary fiber (7.4% on average) combined with overall good sensory acceptability.

## Figures and Tables

**Table 1:** Formulations and processing conditions of the developed whole-meal pasta samples.

Sample	Whole Semolina (SEM) (%)	Fine Bran (FB) (%)	Coarse Bran (CB) (%)	Dough Moisture Content (%)	Hydration and Mixing Duration (Min) in Pre-Mixing Chamber
WP-FB	94	6	0	30	20
WP-CB	90	0	10	30	20

**Table 2:** Ash, protein (N × 6.25), and folate distribution in milling products.

Sample	Moisture (% f.w.)	Ash (% d.m.)	Protein (% d.m.)	Total Folate (μg/100 g d.m.)
DWG	8.3 ± 0.06	1.98 ± 0.021 ^c^	17.4 ± 0.28 ^b^	60.3 ± 1.5 ^c^
SEM	14.4 ± 0.03	1.32 ± 0.049 ^d^	16.7 ± 0.05 ^c^	30.2 ± 2.31 ^d^
FB	13.0 ± 0.07	8.30 ± 0.040 ^a^	22.3 ± 0.08 ^a^	218.9 ± 2.65 ^a^
CB	14.5 ± 0.11	5.43 ± 0.064 ^b^	21.8 ± 0.06 ^a^	204.9 ± 3.82 ^b^

Mean values ± SD. Mean values within a column with different superscript letters indicate statistically significant differences (*p* < 0.05). DWG, durum wheat grain; SEM, semolina; FB fine bran; CB coarse bran.

**Table 3:** Chemical composition, folate, color, and sensorial properties of experimental whole-meal pasta samples.

Pasta Sample	Moisture (g/100 g, f.w.)	Ash (g/100 g, f.w.)	Protein (g/100 g, f.w.)	Fat (g/100 g, f.w.)	Total Dietary Fibre (g/100 g, f.w.)	Carbohydrate * (g/100 g, f.w.)	Total Folate (µg/100 g f.w.)	L*	a*	b*	Furosine (mg/100 g Protein)	OCT (Min)	Total Score	Total Judgement
WP-FB	10.3 ± 0.04 ^a^	1.52 ± 0.014 ^a^	15.0 ± 0.08 ^a^	2.2 ± 0.01 ^a^	6.5 ± 0.29 ^b^	64.4 ± 0.43 ^b^	37.8 ± 1.80 ^b^	52.8 ± 1.12 ^a^	5.2 ± 0.13 ^b^	21.5 ± 0.29 ^a^	224 ± 5 ^b^	15:00	87	More than good
WP-CB	10.0 ± 0.07 ^b^	1.53 ± 0.057 ^a^	15.2 ± 0.09 ^a^	2.4 ± 0.01 ^a^	8.3 ± 0.06 ^a^	62.5 ± 0.29 ^a^	43.1 ± 0.74 ^a^	51.6 ± 1.09 ^a^	6.0 ± 0.27 ^a^	20.7 ± 0.56 ^a^	245 ± 4 ^a^	14:09	82	Good

Mean values ± SD. Mean values within a column with different superscript are statistically significantly different (*p* < 0.05). WP-FB, whole-meal pasta, 94% semolina and 6% fine bran; WP-CB, whole-meal pasta, 90% semolina and 10% coarse bran. * Calculated by difference.

## Data Availability

The data are available from the corresponding author.
